# Study on the efficacy and safety of foldable capsular vitreous body in the severe retinal detachment eyes

**DOI:** 10.1186/s12886-022-02729-9

**Published:** 2022-12-15

**Authors:** Shengnan Ma, Suzhen Zhao, Chunxiao Zhang, Xia Tang, Weiyan Zhou

**Affiliations:** Department of Ophthalmology, Shandong Provincial Hospital Affiliated to Shandong First Medical University, Jinan, 250000 Shandong Province China

**Keywords:** FCVB, Silicone oil, Trauma, Severe retinal detachment

## Abstract

**Background:**

This study was to evaluate the efficacy and safety of the implantation of foldable capsular vitreous body (FCVB) in severe retinal detachment eyes.

**Methods:**

A retrospective study in retinal detachment eyes was performed at Shandong Provincial Hospital Affiliated to Shandong First Medical University. A standard three-port pars plana vitrectomy was performed, and the FCVB was triple folded and implanted into the vitreous cavity. The silicone oil (SO) was then injected into the capsule of the FCVB to support the retina and eye. During the follow-up period, The treated eyes were examined by ophthalmoscopy, fundus photography, and tonometry. B-scan ultrasonography, optical coherence tomography (OCT), and computed tomography (CT), were also performed.

**Results:**

From May 2020 to November 2021, 31 cases with severe retinal detachment were enrolled in the study. The postoperative follow-up time gradient ranged from 1 to 72 weeks, At various observation time points during the 72 weeks after surgery, The postoperative IOP was maintained at around 10 mmhg at various time points, with a slight decrease compared to the preoperative IOP (14.2 ± 4.6 mmHg *n* = 18), and was statistically significant. 9 of 31 patients had clear refractive media, both fundus and OCT showed retinal reattachment, OCT showed the 200 μm thick FCVB capsule support retina. The remaining 22 patients with unclear refractive media, B-scan showed arcuate hyperechoes in front of the retina. There was also no significant difference in visual acuity compared to preoperative. The FCVB was well positioned in the vitreous cavity, and no serious complications such as endophthalmitis, glaucoma, silicone oil emulsification, product exposure, or sympathetic uveitis were found.

**Conclusions:**

FCVB has retinal support with certain ability to maintain IOP and eye morphology and avoid eye removal in patients with severe retinal detachment during the 72-week observation period.

**Supplementary Information:**

The online version contains supplementary material available at 10.1186/s12886-022-02729-9.

## Introduction

The vitreous body is a transparent gelatin structure, which accounts for about 80% of the eyeball. It has the functions of support, refraction, metabolism, and cell barrier. The vitreous body is mainly composed of 95% water and type II glial fibers.At present, common substitutes for vitreous are gas,silicone oil, heavy silicone oil, hydrogels [1–8], among which silicone oil is the most commonly used vitreous substitute for the treatment of complex retinal detachment.

For complex retinal detachment and severe eye trauma, currently the commonly used vitreous replacement is silicone oil. Although the vast majority of patients have been treated, some patients have complications of silicone oil in the treatment process, such as silicone oil emulsification, displacement, glaucoma, cataract, etc. [9–11]. The success rate of the first silicone oil operation was 70% [12], and the success rate of the second silicone oil operation was reduced to 50% [13]. Repeated surgeries caused silicone oil-dependent eyes, low intraocular pressure, and ocular pain. Finally, ocular extraction and implantation of artificial eyes were required for treatment, causing great physical and psychological trauma to patients.

FCVB is a marketed innovative product for the treatment of silicone oil-dependent eyes, it is used to treat severe retinal detachment. It is an intermediate product between silicone oil and artificial eye witch is the last choice for patients without artificial eye. It can support the retina 360 degrees and avoid silicone oil emulsification and displacement. The patient does not need a prone position after FCVB implantation [14–17]. Moreover, in the previous study, because the second-generation specially designed FCVB can well preserve the posterior chamber space, the function of the ciliary body can be slowly restored after the FCVB is implanted [18]. However, FCVB also has shortcomings. Compared with silicone oil, FCVB has a larger surgical incision.

This article summarizes the FCVB study of 31 patients from May 2020 to May 2021 that we performed in Shandong Provincial Hospital Affiliated to Shandong First Medical University.

## Methods

### Subjects

This study protocol was reviewed and approved by the medical ethics committee of Shandong Provincial Hospital Affiliated to Shandong First Medical University (number: 2019–153). The clinical trialsstrictly adhered to the principles of the World Medical Association Declaration of Helsinki. All patients and their families were informed of the study purpose, design and surgical precautions, and voluntarily signed the informed consent for surgery.

The inclusion criteria were as follows: (1) Severe retinal detachment that cannot be treated by current vitreous substitutes; (2) Severe unilateral ocular perforating injuries, compounded retinal or choroidal detachments resulting from retinal rupture or retinal choroidal hemorrhage; (3) Silicone oil cannot be taken out for a long time with incomplete retinal reattachment; (4) Undergone retinal detachment surgery and silicone oil tamponade twice or more.

The exclusion criteria were as follows: (1) Patients are allergic to silicone rubber or scar diathesis; (2) Serious heart, lung, liver or kidney dysfunction, systemic diseases; (3) Uveitis; (4) Other uncontrollable Eye diseases.

### Surgical procedure

Preoperative ophthalmic examination. Including best corrected visual acuity (BCVA), intraocular pressure (IOP), fundus photography, B-ultrasound examination, ocular axis measurement, optical coherence tomography (OCT), computed tomography (CT), and general medical examination. A detailed fundus examination of the contralateral eye was performed to determine the extent of retinal detachment and ocular trauma.

### Procedure for FCVB implantation

**Fig. 1:**
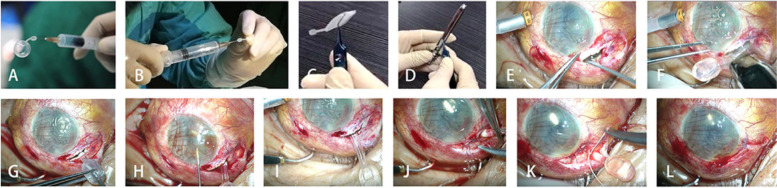
Procedure of FCVB implantation. (1) After conventional vitrectomy, make a peripheral incision under the iris and expand to the supratemporal sclera puncture; (2) Choose the right model and check the tightness of FCVB (see **A**, **B**); (3) The FCVB is folded three times and placed in the injector to be implanted in the eye (see **C**, **D**); (4) The product implantation point is located 5.0 mm behind the scleral limbus of the temporal cornea. A 15° knife is used to cut the sclera vertically. The length of the incision depends on the product model, 3.5–4.5 mm, in an "L" shape(see **E**); (5) Put the product placed in the implanter into the eyeball (see **F**); (6) Inject appropriate silicone oil into FCVB through the drainage valve (see **G**); (7) Inject a proper amount of viscoelastic to restore the anterior chamber (see **H**); (8) The FCVB drainage tube was ligated, fixed on the surface of the iris, and the fascia and bulbar conjunctiva were sutured. (see **J**, **k**); (9) Suture the incision (see **L**)

The operating procedures are shown in Fig. 1 [19] and Video 1: (1) After conventional vitrectomy, make a peripheral incision under the iris and expand to the supratemporal sclera puncture; (2) Choose the right model and check the tightness of FCVB; ( see Fig. 1A, B); (3) The FCVB is folded three times and placed in the injector to be implanted in the eye (see Fig. 1C, D); (4) The product implantation point is located 5.0 mm behind the scleral limbus of the temporal cornea. A 15° knife is used to cut the sclera vertically. The length of the incision depends on the product model, 3.5–4.5 mm, in an "L" shape (see Fig. 1E); (5) Put the product placed in the implanter into the eyeball (see Fig. 1F); (6) Inject appropriate silicone oil into FCVB through the drainage valve (see Fig. 1G); (7) Inject a proper amount of viscoelastic to restore the anterior chamber (see Fig. 1H); (8) The FCVB drainage tube was ligated, fixed on the surface of the iris, and the fascia and bulbar conjunctiva were sutured. (see Fig. 1J, k); (9) Suture the incision (see Fig. 1L).

### Postoperative observation

Postoperative examinations include assessment of the FCVB position, VA through E Standard Logarithm Eyesight, IOP with Goldmann applanation tonometry ( TOPCON, CT-800), slit lamp microscopy (TOPCON, SL-3G), scanning laser ophthalmoscope (SLO) (NIDEK, AFC-330), B-scan (QUANTE, AVISO), optical coherence tomography (OCT) (CRYSTALVUE, FUNDUS VUE), computed tomography scans (SIEMENS SOMATOM FORCE, F6002), as well as monitoring the postoperative complications. The primary effectiveness outcome measure was IOP after FCVB implantation at the 72 weeks follow-up at the latest. The secondary effectiveness outcome measures were retinal reattachment and VA, and the safety outcome was postoperative complications.

## Results

A retrospective evaluation was performed for 31 cases who underwent FCVB implantation at Shandong Provincial Hospital Affiliated to Shandong First Medical University from May 2020 to November 2021. The postoperative follow-up time gradient ranged from 1 to 72 weeks. Of 31 cases, 27 were males and 4 females. The age range was 5–62 years (38.87 ± 15.73). Axis length (AL) of the operative eye ranged from 16.47 to 25.35 mm. 19 of the 31 patients were ocular trauma, 1 of the 31 patients were silicone oil dependent eye, 11 of the 31 patients were ocular trauma and silicone oil dependent eye. The appropriate FCVB size was selected according to the length of the eye axis before operation. 4 sizes of FCVB were used in this study, including 1 case using size AV-10P, 7 cases using AV-12P, 22 cases using AV-13.5P, and 1 case using AV-15P. The average volume of silicone oil injection by 31 cases was 2.6 ± 0.6 ml (i.e. Table 1).

**Table 1:** FCVB size selection, volume of silicone oil and basic information for 31 cases

Case	Age	Gender	Preoperative diagnosis	Size	Retinal reattachment(yes/no)	Volume of Silicone oil (ml)
1	50	M	Left ocular trauma	AV-13.5P	no	2.8
2	49	M	Left ocular trauma	AV-12P	no	1.2
3	56	M	Left ocular trauma	AV-12P	no	1.8
4	38	M	right ocular trauma	AV-13.5P	no	2.5
5	5	F	Left ocular trauma	AV-12P	no	1.8
6	57	M	right ocular trauma	AV-13.5P	no	2.5
7	28	M	Left ocular trauma, silicone oil dependent eye	AV-13.5P	yes	2.8
8	12	M	right ocular trauma	AV-12P	no	1.9
9	52	M	Left ocular trauma, silicone oil dependent eye	AV-13.5P	yes	3.0
10	31	M	Right ocular trauma	AV-13.5P	no	3.0
11	30	M	Left ocular trauma	AV-12P	no	1.7
12	51	M	Right ocular trauma, silicone oil dependent eye	AV-13.5P	yes	3.1
13	57	M	right ocular trauma	AV-10P	no	1.2
14	24	M	Left ocular trauma	AV-12P	no	2.0
15	62	M	right ocular trauma	AV-13.5P	no	2.9
16	32	M	Left ocular trauma, silicone oil dependent eye	AV-13.5P	yes	3.0
17	6	F	Silicone oil dependent eye	AV-13.5P	yes	2.8
18	62	M	Left ocular trauma	AV-13.5P	no	2.8
19	32	F	Right ocular trauma, silicone oil dependent eye	AV-13.5P	no	3.0
20	43	M	Right ocular trauma, silicone oil dependent eye	AV-13.5P	yes	3.0
21	40	M	Left ocular trauma, silicone oil dependent eye	AV-13.5P	no	2.0
22	43	M	Left ocular trauma	AV-13.5P	no	3.0
23	36	M	Left ocular trauma, silicone oil dependent eye	AV-13.5P	yes	3.0
24	31	M	Left ocular trauma	AV-13.5P	no	2.5
25	43	M	right ocular trauma	AV-13.5P	no	2.4
26	55	M	Right ocular trauma, silicone oil dependent eye	AV-12P	no	2.8
27	46	F	Left ocular trauma	AV-13.5P	yes	3.0
28	33	M	Right ocular trauma, silicone oil dependent eye	AV-13.5P	yes	2.8
29	55	M	Left ocular trauma	AV-13.5P	no	3.0
30	30	M	Right ocular trauma, silicone oil dependent eye	AV-15P	no	4.0
31	16	M	Left ocular trauma	AV-13.5P	no	3.0

### Effectiveness analysis of FCVB implantation

**Fig. 2:**
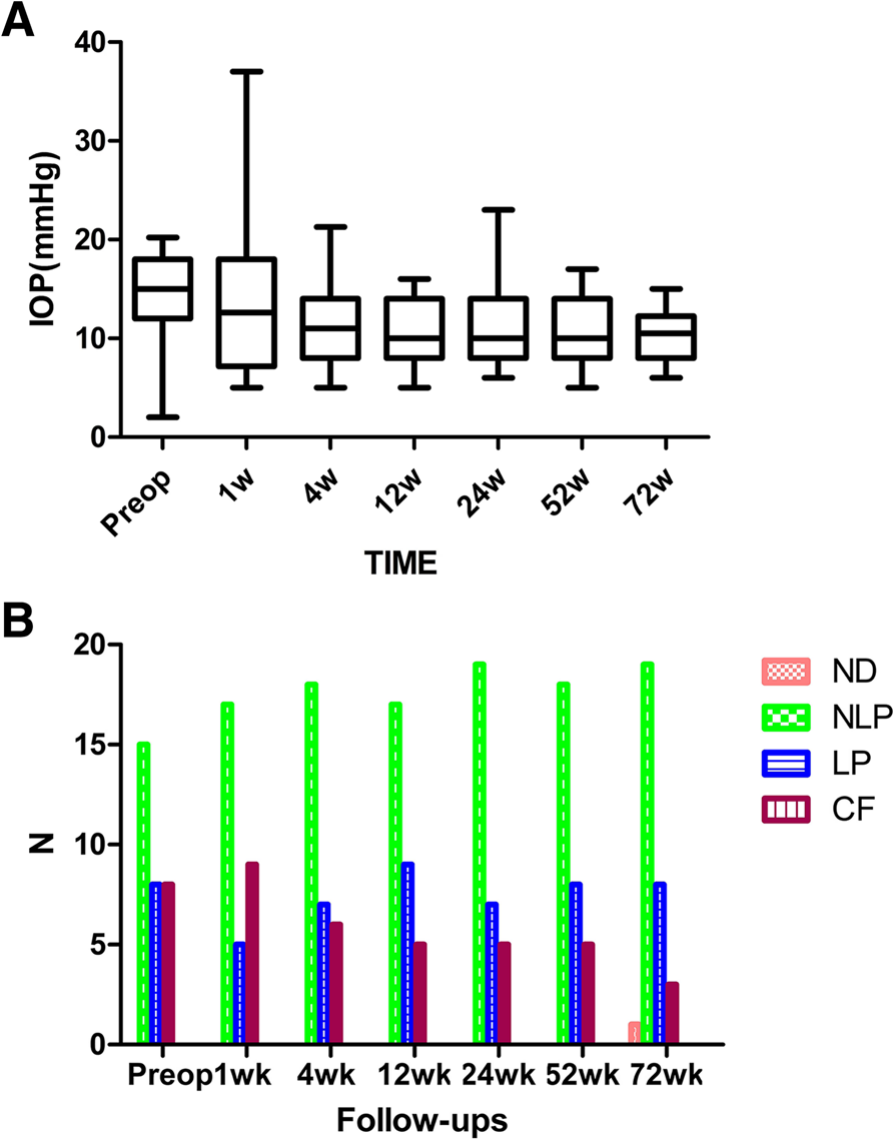
**A** The intraocular pressure scores of the patients within 72 weeks were statistically analyzed. **B** The visual acuity scores of the patients within 72 weeks were statistically analyzed

Main endppints. At various observation time points during the 72 weeks after surgery, The postoperative IOP was maintained at around 10 mmhg at various time points, with a slight decrease compared to the preoperative IOP (14.2 ± 4.6 mmHg *n* = 18), and was statistically significant. The IOP at 1 week, 4 weeks, 12 weeks, 24 weeks,52 weeks, 72 weeks, after surgery was 12.6 ± 7.4 mmHg (*n* = 18,*P* = 0.396), 10.5 ± 4.2 mmHg (*n* = 18,*P* = 0.010), 10.0 ± 3.1 mmHg (*n* = 18,*P* = 0.001),, 10.1 ± 3.3 mmHg (*n* = 18,*P* = 0.003), 10.1 ± 3.3 mmHg (*n* = 18,*P* = 0.002) and 9.8 ± 2.7 mmHg (*n* = 18,*P* = 0.001), respectively, as show in Fig. 2A.

**Fig. 3:**
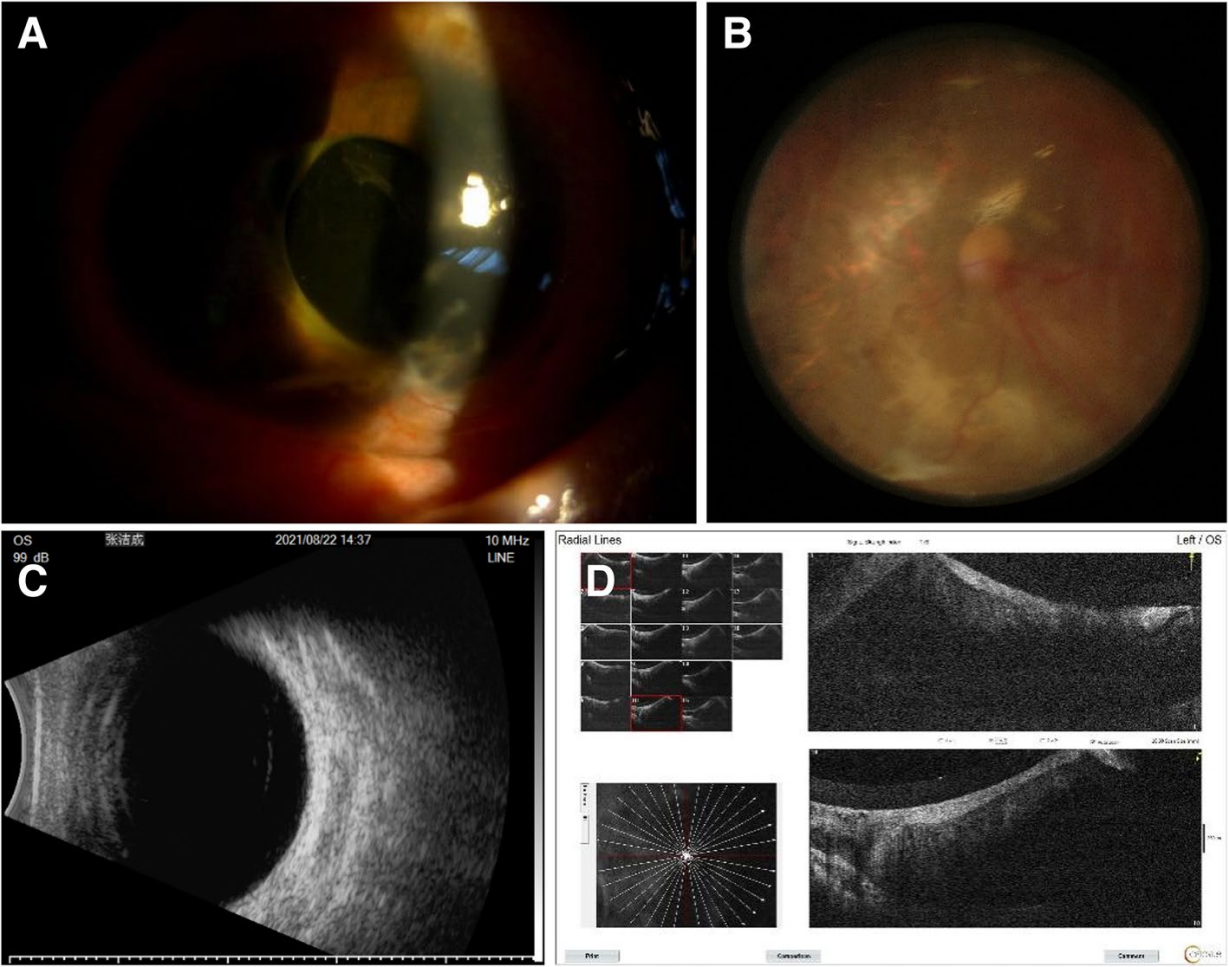
Examination of Case 7 after 1-year-implantation of FCVB. **A** Anterior segment showed there is a deep anterior chamber. **B** Fundus photography six months after the operation, the retina was reset. **C** The B-scan showed that a capsule-like arc reflective signal was supporting the retina. **D** OCT showed that the contact between the FCVB and retina was smooth

**Fig. 4:**
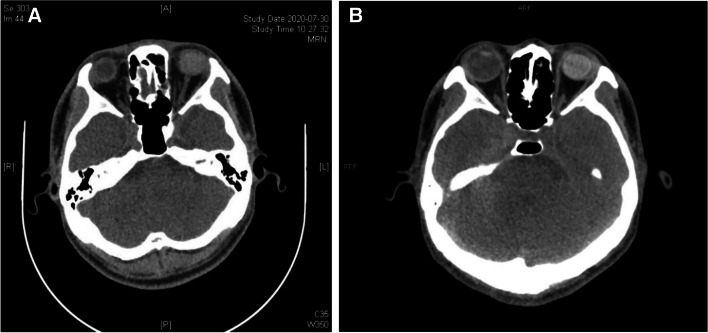
Postoperative follow-up results of FCVB implantation in case 20. **A** Preoperative CT. **B** CT 24 Weeks after operation

Secondary end points. 9 of 31 patients had clear refractive media, both fundus and OCT showed retinal reattachment, OCT showed the 200 μm thick FCVB capsule support retina. The FCVB was observed to be properly positioned in the vitreous cavity and providing adequate support to the retina (Fig. 3A, B, D). The remaining 22 patients with unclear refractive media, B-scan showed arcuate hyperechoes in front of the retina (Fig. 3C). Horizontal CT showed that the distended FCVB could support the posterior part of the eye well with some depth of anterior and posterior chamber space (Fig. 4). The visual acuity at 72 weeks of postoperative follow-up is summarized in Fig. 2B and Supplementary Table 1. The number of cases with no light perception remained essentially the same after surgery compared to the preoperative period, with a slight decrease in the number of count fingers patients, but visual acuity results were not statistically significant.

### Safety analysis of FCVB implantation

Implantation of the FCVB was accomplished according to standard operating procedures in all patients, with no cases of failure to implant. Of the 31 cases, 3 exhibited corneal opacity without keratopathy, and 4 exhibited a shallow anterior chamber. although 2 patients had mild hemorrhage caused by previous severe ocular damage. The FCVB was well positioned in the vitreous cavity, and no serious complications such as endophthalmitis, glaucoma, silicone oil emulsification, product exposure, or sympathetic uveitis were found.

### Representative case 07: follow-up for 52 weeks

No serious complications were observed in this patient up to 52 weeks, and post-operative ophthalmoscopy, fundus photography, B-ultrasound and OCT at 52 weeks indicated good status, and the retina was reattachment (see Fig. 3), The FCVB full of SO in the eyeball can be observed clearly on horizontal CT, FCVB could support the posterior part of the eye well with some depth of anterior and posterior chamber space (Fig. 4). 

### Representative case 26: follow-up for 24 weeks

**Fig. 5:**
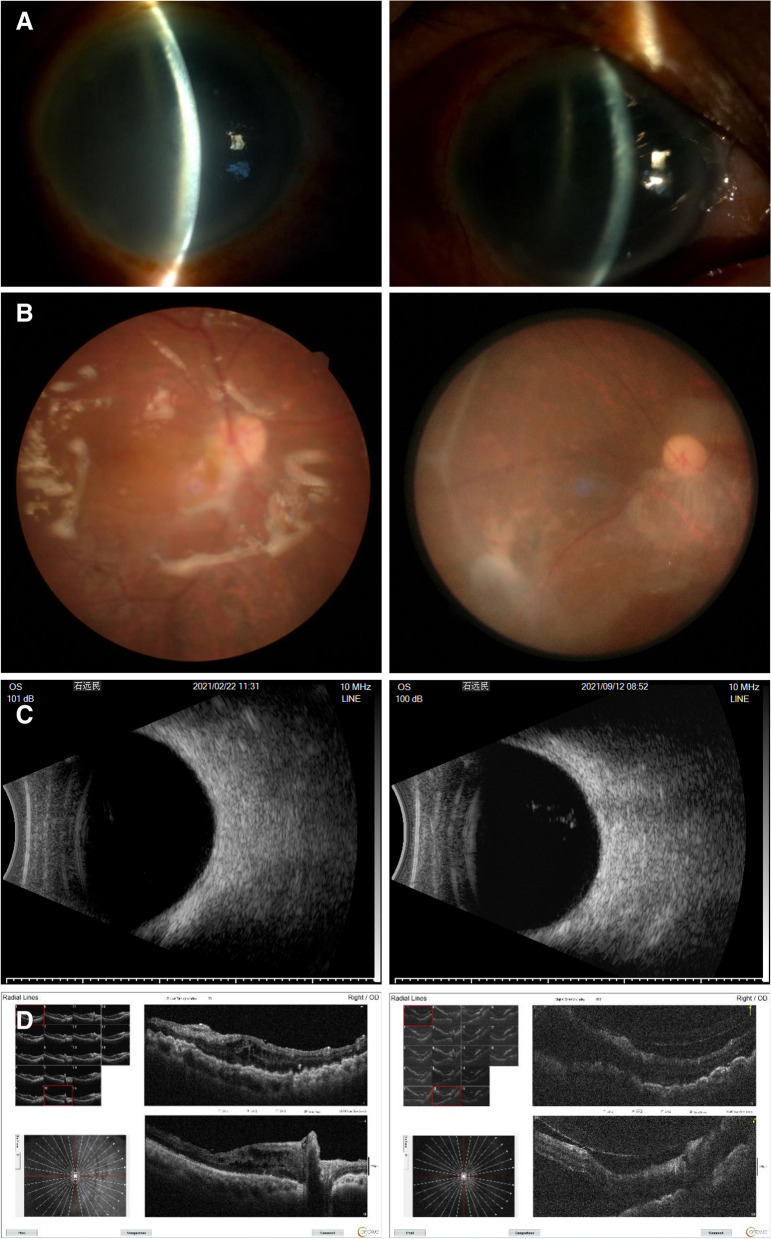
Examination of Case 26 after 24 weeks implantation of FCVB. **A** Preoperative and 4 week postoperative photographs of the anterior segment of the eye showed clear corneas and deep anterior chamber in the operated eye; **B** Preoperative and 24 weeks postoperative fundus photographs showed retina has not repositioned; **C** The B-scan showed that a capsule-like arc reflective signal was supporting the retinal **D** OCT showed that the contact between the FCVB and retina was smooth

This case has completed 24 weeks of post-operative follow-up and no serious complications were observed. The patient's recovery was satisfactory according to a comparison of the preoperative and postoperative findings, which are shown in Fig. 5.

## Discussion

This study involved the evaluation of 31 patients with severe ocular trauma and SO dependent eyes who under-went a 23G PPV combined with FCVB implantation and SO tamponade from May 2020 to November 2021. The study show that FCVB implantation can preserve the eyeball and provide adequate support for the retina, and it has certain effect on retinal reattachment in severe retinal detachment eyes.

Nowadays several vitreous substitutes such as inert gas, SO, heavy SO, hydrogel, etc. are used in clinic [20–25]. SO has become the most important and effective vitreous substitute in the past 50 years since SO was used as vitreous substitute by Cibis in 1962 [1]. But SO still has many defects, especially the poor long-term security compared to natural vitreous. A series of complications due to long-term intraocular SO tamponade may occur such as cataracts, corneal lesions, glaucoma, etc. [9, 26–30]. In addition, SO can lead to great damage of retina and optic nerve as reported in past years [31–33]. The SO emulsification ratio can even be 100% when SO remains in the eye for 1 year according to research [28]. Thus, the safety of vitreous substitutes need to pay much more attention.

FCVB as an innovative vitreous substitute product is deemed potential in the future, which is made of inert medical silicone. SO is injected into the capsule through valve in the treatment, thus it is not easily reacted in the eye, avoiding emulsification, translocation and other complications [14]. FCVB shape is very similar with natural vitreous obtained by computer simulation of natural vitreous cavity shape, which can 360-degree support retina [34]. For patients with severe eye trauma or SO-dependent eyes, the primary purpose of treatment is to maintain eyeball shape, avoid eyeball atrophy and eventually restore some vision. The lens plane of the second-generation FCVB is flat, which can keep the anterior and posterior chambers, avoid silicone oil penetration into the ciliary body, avoid damage to the ciliary body, and allow it to resume its basic function and aqueous humor secretion.

As mentioned in the above article, silicone oil-dependent eyes are prone to many complications, and The SO emulsification ratio is high. In the past, the treatment of silicone oil-dependent eyes required multiple surgeries or even enucleation. In recent years, the treatment of silicone oil-dependent eyes is by FCVB implantation. The safety and efficacy of FCVB for silicone oil dependent eyes were also supported in the recent study [11, 14, 19]. FCVB is a new potential treatment for severe silicone oil-dependent eyes, which can maintain ocular morphology without severe complications [35].

To sum up, in severe retinal detachment treatment, FCVB implant is an effective new operation method, can control the eyeball atrophy, part of patients achieved retinal reattachment, and no severe complications, and some literature shown that it can also eliminate the psychological barriers, improve patient satisfaction, have great potential for clinical application [36–39].

This study involved the evaluation of 31 patients underwent a 23G PPV combined with FCVB implantation and SO tamponade from May 2020 to November 2021. 9 of 31 patients had clear refractive media, both fundus and OCT showed retinal reattachment. Compare with other studies, the rate was similar to Zhang and Tian’s study [14], which was 20 patients with severe ocular trauma or silicone oil (SO) dependent eyes, and 6 of them achieved retinal reattachment 12 months after FCVB implantation, the reattachment rate was 30% either.

For the 31 cases who experienced decreased visual acuity and decreased intraocular pressure, the following reasons were summarized separately.

### Causes of decreased visual acuity

1. Factors of the surgery itself, the surgical procedure involves the reset of the retina, which inevitably has some impact on the retina; 2. Factors of the design principle of FCVB, FCVB is a transparent capsule membrane, but the design level does not reach the effect of invisibility, which will still have a slight impact on the vision.

### Causes of IOP drop

1. FCVB location factor, FCVB is located in the posterior segment of the eye, which has a parietal effect on the retina, but no direct parietal effect on the cornea, iris, anterior segment IOP decrease; 2. The patient's own ciliary body is damaged, the incision is located in the flat part of the ciliary body, which again affects part of the ciliary body function, causing IOP decrease.

## Conclusions

The finding presented that FCVB implantation can preserve the eyeball and provide adequate support for the retina, it also has certain effect on retinal reattachment in severe retinal detachment eyes during the 72 Weeks implantation period. And the FCVB was well distributed in the vitreous cavity and evenly supported the retina.

## Supplementary Information

**Additional file 1: Supplementary Table 1.** Visual acuity after FCVB implantation during 72-week observation time.

**Additional file 2: Video 1.** FCVB implantation.

## Data Availability

All data generated or analysed during this study are included in this published, The relevant raw data will be freely available from the corresponding author upon request.
