# Supplementary material for: Study on the efficacy and safety of foldable capsular vitreous body in the severe retinal detachment eyes
Source: BMC Ophthalmol. 2022 Dec 15;22:491. doi: 10.1186/s12886-022-02729-9 (PMC9756693; doi:10.1186/s12886-022-02729-9)
Supplement: Supplementary file 1 — Additional file 1: Supplementary Table 1. Visual acuity after FCVB implantation during 72-week observation time. [file 12886_2022_2729_MOESM1_ESM.docx]

Supplementary Table 1. Visual acuity after FCVB implantation during 72-week observation time.

| Follow-ups | ND (*n*) | NLP (*n*) | LP (*n*) | CF (*n*) |
| --- | --- | --- | --- | --- |
| Preop | 0 | 15 | 8 | 8 |
| 1wk postop | 0 | 17 | 5 | 9 |
| 4wk postop | 0 | 18 | 7 | 6 |
| 12wk postop | 0 | 17 | 9 | 5 |
| 24wk postop | 0 | 19 | 7 | 5 |
| 52wk postop | 0 | 18 | 8 | 5 |
| 72wk postop | 1 | 19 | 8 | 3 |

ND:No Done;NLP: No light perception; LP: Light perception; CF: Count fingers; IOP: Intraocular pressure.
